# Partial characterization, antioxidative properties and hypolipidemic effects of oilseed cake of *Allanblackia floribunda* and *Jatropha curcas*

**DOI:** 10.1186/1472-6882-13-352

**Published:** 2013-12-11

**Authors:** Thaddée Boudjeko, Judith Emery Kanemoto Ngomoyogoli, Alice Louise Woguia, Nicolas Njintang Yanou

**Affiliations:** 1 Laboratory of Phytoprotection and Valorisation of Plants Resources, Biotechnology Centre - Nkolbisson, P.O. BOX 3851, Messa, Yaounde, Cameroon; 2 Department of Biochemistry, Faculty of Science, University of Yaounde I, P.O. BOX 3851, Messa, Yaounde, Cameroon; 3 Department of Biological Sciences, Faculty of Science, University of Ngaoundere, P.O. Box 454, Ngaoundere, Cameroon

**Keywords:** *A. floribunda*, *J. curcas*, Oilseed cake, Antioxidant and scavenging activities, Lipid profile

## Abstract

**Background:**

High fat diet is known to induce oxidative stress and abnormal changes in lipid metabolism. Many traditional plants have been shown to possess antioxidant and lipid-lowering activities, improving on oxidative status and lipid profile. In this paper, we characterized and examined the antioxidative properties of the oilseed cake of *A. floribunda* and *J. curcas*. We also evaluated their effect on lipid profile in the plasma and liver of experimental rats placed on a high fat diet.

**Methods:**

For a partial characterization, the qualitative and quantitative analyses of storage proteins, dietary fibre and polyphenol content were evaluated. Four extracts (aqueous, ethanolic, methanolic and 0.1 N HCl) were evaluated for their antioxidant properties and scavenging activities. The effect on lipid profile was evaluated after the administration of the crude extracts to albino rats placed on a high fat diet.

**Results:**

Our results showed that *J. curcas* contains 10 times more storage proteins than *A. floribunda* while *A. floribunda* contains twice as much total dietary fibre than *J. curcas*. An evaluation of the different families of storage proteins showed that *J. curcas* has glutelins as the major storage proteins in its seeds (61.65 mg/g d.m), followed by globulins (25.30 mg/g d.m) and albumins (18.30 mg/g d.m). The electrophoretic analyses revealed a diversity of bands at the level of the different families and for both species. The evaluation of the *in vitro* antioxidant activities showed that *A. floribunda* extracts had higher antioxidant properties. Although the composition of *A. floribunda* and *J. curcas* oilseed cake are different, they lowered serum triglycerides (TG), total cholesterol (TC) and blood glucose level.

**Conclusion:**

These results show that the oilseed cake of *A. floribunda* and *J. curcas* possess antioxidant properties with an effect on blood glucose level and lipid profile.

## Background

Hypercholesterolemia and hypertriglyceridemia are major risk factors that, alone or together, can accelerate the development of coronary artery diseases and the progression of atherosclerotic lesions [1]. Normally, the major part of cholesterol serves as structural element in the cell membranes, whereas much of the rest is in transit through the blood or functions as the starting material for the synthesis of bile acids in the liver, steroid hormones in endocrine cells, or vitamin D in the skin. However, high levels of cholesterol accumulate in the extracellular subendothelial space of arteries and are modified to form oxidized LDL, which is highly atherogenic and toxic to vascular cells [2]. LDL levels in blood have been associated to a variety of chronic diseases such as atherosclerosis, hypertension, obesity, diabetes, functional depression of some organs, etc.…. In addition, there is a relationship between hyperlipidemia (hypercholesterolemia) and the increased production of oxygen free radicals and the antioxidative defense system [3,4]. In fighting against metabolic disorders, some naturally occurring compounds with antioxidative properties may have beneficial effects on the overall disease processes [5]. *Jatropha curcas and Allanblackia floribunda* are two tropical plants shrubs which fall within this scope.

*Jatropha curcas* L. (physic nut, purging nut or pig nut), belonging to the family of Euphorbiaceae, is currently used worldwide industrially for the production of biodiesel. Besides, it is also used in traditional folklore medicine to cure various ailments in Africa, Asia and Latin America [6,7]. The crude ethanolic extract of the leaves has shown antimicrobial properties against many bacteria including Staphylococcus spp., Streptococcus spp. and E. coli [8]. The water extract of the branches strongly inhibits HIV induced cytopathic effects with low cytotoxicity [6]. The leaves of *J. curcas* contain apigenin, vitexin and isovitexin which along with other factors enable them to be used against malaria, rheumatic and muscular pains [9].

*Allanblackia floribunda* Oliver, commonly known as tallow-tree or ouotera, is a member of the mangosteen family (Guttiferae Juss. 1789 vs. Clusiaceae Lindl. 1836). The bark of *A. floribunda* is used against cough, dysentery, diarrhea, toothache, and is an analgesic [10]. The stem bark extract possesses aphrodisiac, antihypertensive and antioxidant properties [11]. Moreover, the seeds are rich in a hard white fat (67–73%) consisting mostly of stearic and oleic acids [12]. Oleic and stearic acids are reported to lower plasma cholesterol levels [13], thus reducing the risks of heart attack. Owing to this property, *A. floribunda* seed fat is used for margarine production and in the manufacture of soap and ointments [14].

Whatever limited information available on the medicinal properties of *J. curcas* and *A. floribunda* is mostly on the leaf extracts, latex, oil or stem bark of the plant. In this paper, we examined the antioxidative properties of oilseed cake of *A. floribunda* and *J. curcas*. We also evaluated their effect on lipid profile in the plasma and liver of experimental rats placed on a high fat diet.

## Methods

### Plant material

Dry seeds of *A. floribunda* (Voucher N: 1380/CNH) and *J. curcas* (Voucher N: 25713/CNH) were collected from the Centre and North regions of Cameroon respectively in September 2010 and authenticated by the Cameroon National Herbarium. Upon collection, the identities of the plants were determined by the Cameroon National Herbarium in Yaounde. The dried seeds were finely ground and defatted with hexane by exhaustion.

### Proteins extraction and analyses

Storage protein fractions were extracted from defatted powder according to Nasri and Triki [15]. 15 mg of residue were mixed with 1 mL distilled water at 4°C for 1 hr and then centrifuged at 10000 g for 20 min at the same temperature. The supernatant containing albumins was collected, while the pellet was used in further extractions. In this respect the pellet was rinsed with 0.5 mL distilled water before a 30 min homogenization, followed by centrifugation under the same conditions as in the previous step, to remove albumins completely. The pellet obtained, underwent a similar series of steps (homogenization-centrifugation, rinsing) using a mixture of 100 mM Tris HCl in 0.5 M NaCl at pH 8.1 to extract globulins. The second pellet was submitted to a third and similar extraction of prolamins in 70% ethanol, and glutelins in acetic acid 0.2 N. The four protein groups obtained were quantified by the Bradford method [16]. The protein bands were then determined using SDS-PAGE (12%, pH 8.8) according to the method of Laemmli [17]. The estimation of molecular weight was done based on the Pre-stained Protein Marker, Broad Range P7708S. At the end of the migration, gels obtained were immerged for 2 hrs, in a staining solution made up of methanol/acetic acid/distilled water (50/10/40, v/v/v) and 0.25% Coomassie Brillant Blue R-250. After staining, the gel with protein bands were snapped using a numeric photo apparatus (Samsung) to produce the electrophoregrammes.

### Total dietary fibres content

Total dietary fibre content was estimated using a modified AOAC 2000 [18] method. 2 g of defatted powder were added to 10 mL of α-amylase (Sigma Chemical Co. Ltd) 2% in phosphate buffer 0.1 M pH 7 and protease. Then, the residue was rinsed with 20 mL of boiling water and a cold solution of amyloglucosidase (EC: 3.2.1.1 Megazyme International) was added. A solution of 80% ethanol was added and the mixture filtered and rinsed with boiling water. The residue was weighted and used for the quantification of ashes and proteins.

### Antioxidant properties

The antioxidant properties of the defatted powders were evaluated on different solvent extracts: water, ethanol (70%), methanolic (80%) and 0.1 N HCl, as described by Dicka et al. [19]. One gram of defatted powders was extracted in 20 ml of respective solvents in a Soxhlet apparatus for 24 h. The extracts were centrifuged and filtered for antioxidant properties.

### Total phenolic content

The amount of total phenolic compounds in extracts was determined using Folin–Ciocalteu reagent, according to the method of Singleton and Rossi [20] with some modifications and using ferulic acid as a standard. Briefly, 500 μL of extract solution were added to 1500 μL of distilled water then 75 μL of Folin-Ciocalteu reagent and mixed thoroughly. After 3 min, 750 μL Na_2_CO_3_ (20%) were added. The mixture was allowed to stand for 1 h with intermittent shaking. The absorbance was measured at 760 nm using a visible spectrophotometer. The total phenolic content was expressed as mg of ferulic acid equivalent per g of dry matter, using a standard curve prepared using ferulic acid.

### Ferric reducing antioxidant potential (FRAP) assay

The ferric reducing power of plant extracts was determined using a modified Benzie and Strain [21] method. Essentially the FRAP reagent containing 1 volume of 10 mmol/L TPTZ (2, 4, 6- tripyridyl- s- triazine) solution in 40 mmol/L HCl, 1 volume of 20 mmol/L FeCl_3_ and 10 volume of 0.3 mol/L acetate buffer (pH = 3.6) was prepared freshly. Aliquots of 100 μL extract (1000 μg/mL) were mixed with 2 mL FRAP reagent and the absorbance of the reaction mixture at 593 nm was measured spectrophotometrically after incubation at 37°C for 12 min. For construction of the standard curve, 5 concentrations (1000, 750, 500, 250, 125 μmol/L) of FeSO_4,_ 7H_2_O were used and the optical densities were measured as sample solution. The values were expressed as the concentration of antioxidants having a ferric reducing ability equivalent to that of FeSO_4_.

### DPPH free-radical-scavenging assay

The method described by Katalinie et al*.*[22] was adopted. To 100 μL of variable concentrations of the extract (100; 250 and 500 μg/mL) was added 500 μL DPPH solution (400 μM in methanol). The mixture was stirred and left in the dark for 30 min. The absorbance was measured at 517 nm using UV-1605 Shimadzu spectrophotometer and ascorbic acid was used as the positive control.

### ABTS free-radical-scavenging assay

The method described by Re et al. [23] was adopted. To 100 μL of variable concentration of extract (100; 250 and 500 μg/mL) was added ABTS reagent. The mixture was stirred and left in the dark for 30 min. The absorbance was measured at 734 nm using UV-1605 Shimadzu spectrophotometer and ascorbic acid was used as the positive control.

### Chelating ability for ferrous ions

The method of Dinis et al. [24] was used to determine the ferrous ion chelating ability. 100 μL of different extracts (100; 250 and 500 μg/mL) were mixed with 100 μL of 2 mM FeCl_2_ and left in the dark for 30 s. The mixture was added to 200 μL of 5 mM ferrozine. After 10 min at room temperature, the absorbance of the mixture was determined at 562 nm against a blank. A lower absorbance indicated a higher chelating power and EDTA was used as the positive control.

### *In vivo* study of hypolipidemic effect of defatted products

Investigations on animals were conducted in accordance with the internationally accepted principles for laboratory animal use and care as found in the United States guidelines (United States National Institutes for Health publication n° 85–23 revised in 1985). The animals were maintained under standard laboratory conditions with 12 h light and dark cycle, with free access to standard laboratory rat food and tap water (temperature of 24 ± 1°C and humidity of 55 ± 10%). Prior authorization for the use of laboratory animals in this study was obtained from the Cameroon National Ethical Committee.

Twenty male Wistar rats (180–210 g) bred in the laboratory were randomly divided into four groups of five rats each and then housed separately in partitioned polypropylene cages labeled as control, high fat diet control, high fat diet treated + 250 mg.kg^-1^*A. floribunda* extract*;* high fat diet treated + 250 mg.kg^-1^ *J. curcas* extract. The rats were allowed free access to water and feed (diet) *ad libitum* for 15 days under controlled environmental conditions of temperature and relative humidity and a 12-hour light and dark cycle. Treatment was administered daily by force-feeding with water for control group and high fat diet group, 100 mg. mL^-1^ of aqueous crude extract of *A. floribunda* or *J. curcas* at dose of 250 mg per kg body weight of experimental animals respectively. The control group was fed the normal diet while the high fat diet treated rats were fed with high fat diet, containing 23 g of casein, 5 g of cellulose, 10 g of sucrose, 46 g of starch, 10 g of lard, 4 g of corn oil and 0.15 g of margarine.

### Sample collection

On the last day of the experiment, rats were deprived of food for 12 h and then anaesthetized by ether inhalation and sacrificed by decapitation. Blood was collected from the heart into EDTA tubes. Plasma was prepared by centrifugation at 3,000 rpm for 15 minutes (Clay-Adams Co. Inc. centrifuge, New York, USA) and used for the estimation of lipid profile. Livers from the animals were rinsed in ice-cold 1.15% KCl, dried and weighed. It was homogenized in 4 volumes of ice-cold 50 mM phosphate buffer, pH 7.4, and centrifuged at 6,000 rpm (Clay-Adams Co. Inc. centrifuge, New York, USA) for 20 min to obtain post mitochondrial fraction (PMF). Total cholesterol (TC) and triglycerides (TG) were determined enzymatically using commercially available kits from Chronolab and blood glucose level was measured after 18 hrs of fasting on day 0 and day 14.

### Statistical analyses

The results were expressed as mean ± standard deviation of three parallel measurements; the Kruskal Wallis test was used to evaluate the significance of the variation amongst the test group, and when this was verified, a Least Significant Difference (LSD) test was used to compare two means. The level of significance was set at *p* < 0.05 and the graphical representations were designed in Microsoft Excel 2007.

## Results

### Protein extraction and analysis

**Figure 1:**
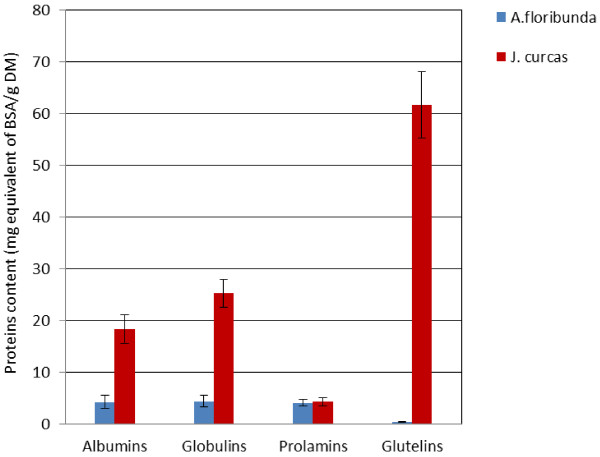
**Storage proteins content in *A. floribunda *** **and *J. curcas *** **seeds.**

The composition of proteins showed considerable differences between the two species (Figure 1). In general, the total protein content was higher in *J. curcas* than *A. floribunda.* Glutelins (61.65 mg/g dry mass (d.m)) were the predominant proteins in *J. curcas* followed by globulins (25.30 mg/g d.m) and albumins (18.30 mg/g d.m). However, in *A. floribunda* seeds globulins (4.37 mg BSA/g d.m), albumins (4.24 mg BSA/g d.m) and prolamins (4.12 mg BSA/g d.m) had similar amounts.

**Figure 2:**
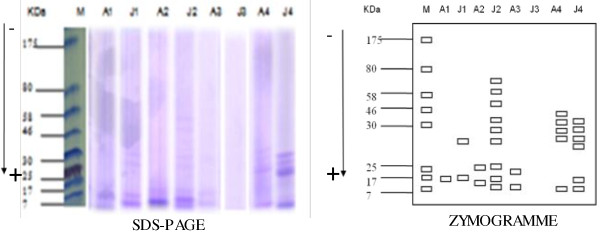
**Different sub-units of storage proteins.** a = electrophoregramme; b = zymogramme; M = molecular marker; A = *A. floribunda*; J = *J. curcas*; 1 = albumin; 2 = globulin; 3 = prolamin; 4 = glutelin.

The electrophoretic analysis by SDS-PAGE showed bands with molecular weight ranging from 7 to 64 kDa (Figure 2). *J. curcas* had the highest diversity in globulin sub-types, with 9 bands (7.3; 8.43; 14.79; 25.47; 27.18; 29.85; 47.8; 54.3 and 64 kDa), followed by glutelins, 6 bands (7; 8.3; 25.4; 25.7; 26.31; and 27.58 kDa). *A. floribunda* contained globulins with only two sub-units (9 and 15 KDa), albumins with one sub-unit of 13.4 KDa, prolamins with two bands (7.03 and 14.12 kDa) and glutelins with five bands (7; 25.71; 26.31; 27.58 and 29.19 KDa) (Figure 2).

### Total dietary fibres

Total dietary fibres estimated by enzymatic procedure were higher in *A. floribunda* (49.57%) than *J. curcas* (24.47%).

### Total phenolic content and FRAP antioxidant capacities

The total phenolics compounds and FRAP antioxidant capacity of *A. floribunda* and *J. curcas* extracts obtained from various solvents are presented in Table 1. Generally, ethanol extracted more phenolic compounds than methanol and water (Table 1). The difference between *J. curcas* and *A. floribunda* was observed only with the methanolic extract which was significantly higher in *A. floribunda* than *J. curcas*. On the contrary, the FRAP antioxidant capacity was systematically higher in all extracts of *A. floribunda* than *J. curcas* except HCl. The ethanolic extract was ranked first (21.80 ± 0.50 mg/g d.m) followed by methanolic (15.82 ± 0.48 mg/g d.m) and aqueous (11.02 ± 0.08 mg/g d.m) extracts (Table 1).

**Table 1:** Total phenolic content and FRAP antioxidant capacities

**Extracts**	***A. floribunda***	***J. curcas***
**Total phenolic content (mg equivalent of ferulic acid/g d.m)**		
Aqueous	47.17 ± 2.01^a^	43.93 ± 2.98^a^
Ethanolic	62.69 ± 7.20^a^	66.64 ± 0.12^a^
Methanolic	54.39 ± 0.20^b^	31.54 ± 2.39 ^a^
HCl 0.1 N	15.38 ± 0.56^a^	18.8 ± 0.01^a^
**FRAP antioxidant capacities (mg equivalent of ascorbic acid/g d.m)**		
Aqueous	11.02 ± 0.08^b^	5.03 ± 0.42^a^
Ethanolic	21.80 ± 0.50^b^	7.70 ± 0.44 ^a^
Methanolic	15.82 ± 0.48^b^	2.85 ± 0.55 ^a^
HCl 0.1 N	3.72 ± 0.04^a^	3.85 ± 0.01 ^a^

Data are presented as mean ± SD (n = 5); mean values within a line with different superscript letters are statistically different (*p* < 0.05). d.m: dry mass.

### DPPH and ABTS free-radical-scavenging assay

**Figure 3:**
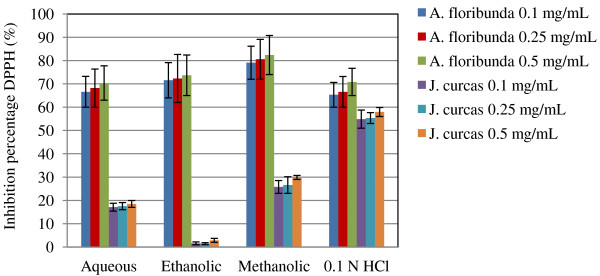
**DPPH radical scavenging activity in different extracts of seeds oil-cake of *A. floribunda *** **and *J. curcas*.**

**Figure 4Figure 4:**
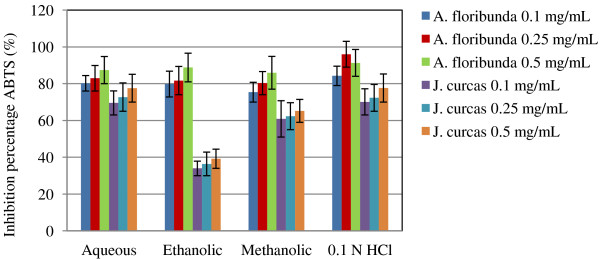
**ABTS radical scavenging activity in different extracts of seeds oil-cake of *A. floribunda *** **and *J. curcas*.**

The results showed that the DPPH scavenging capacity (Figure 3) of the *A. floribunda* extract was higher than that of the *J. curcas* and increased with increasing extract concentrations. Moreover, *A. floribunda* had the highest DPPH scavenging activity in the methanolic fraction, while in *J. curcas* the highest activity was obtained with the 0.1 N HCl fraction. With ABTS, the inhibition percentage fluctuated between 78.9 ± 0.98% and 80.2 ± 0.05% for *A. floribunda* while in *J. curcas* it fluctuated between 11.8 ± 1.65% and 69.5 ± 2.44%. Irrespective of the plant nature, the aqueous extract had the highest (Figure 4).

### Chelating capability for ferrous ions

**Figure 5:**
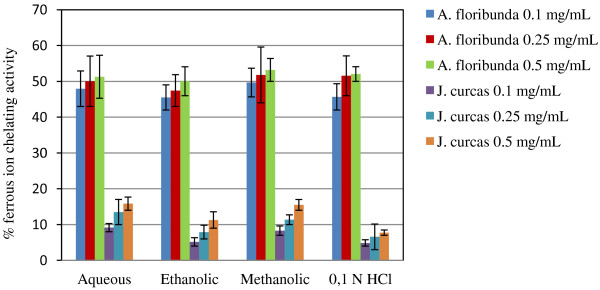
**Iron metal ions chelating activity in different extracts of seeds oil-cake of *A. floribunda *** **and *J. curcas*.**

Metal (Fe2+) chelating activity (Figure 5) showed that *A. floribunda* at the concentrations tested (0.1, 0.25 and 0.5 mg/ mL) had a larger percentage of metal-chelating capacity than *J. curcas.*

### Food intake, weight gain, serum lipid level and hypoglycemic activity

Hyperlipidemia and hyperglycemia were induced by administering high fat diet to rats. The administration of high fat diet to the rats affected the mean food intake and body weight gain of the animals in different ways depending on the experimental groups (Table 2). In the animals fed with high fat diet, *A. floribunda* and *J. curcas* led to significant (*p* < 0.05) decrease in total food intake as compared to the controls. Moreover, the body weight varied significantly (*p* < 0.05) between the groups at the end of the 14-day experience. Weight gain throughout the high-fat treatment was significantly (*p* < 0.05) lower in the animals that received *A. floribunda* and *J. curcas* than in those that received the high-fat diet only (Table 2). To investigate the effect of the oilseed cakes of *A. floribunda* and *J. curcas* on the lipid levels of rat serum and the post mitochondrial fraction (PMF) of liver, the amounts of total cholesterol (TC) and triglycerides (TG) were measured. The results showed that rats fed with high fat diet in the absence of the plant extracts had their amounts of TG and TC in plasma increased by 31.7% and 47.2% respectively compared to the control, while in the liver, the increase was 39.0% and 41.0% (Table 3). The amounts of plasma TC and TG significantly (*p* < 0.05) decreased after the administration of oilseed cake of *A. floribunda* and *J. curcas*. For instance, the levels of plasma TC and TG reduced by 13.06% and 30.52% respectively for rats that received *A. floribunda*; and 18.57% and 13.42% for those that received *J. curcas* compared to the high fat diet group (Table 3). However, in the post mitochondrial fraction (PMF) of the liver, the amounts of TC significantly decreased after administration of oilseed cake while the amounts of TG increased compared to the control group (Table 3). The induced hyperglycaemia, caused by administering high fat diet to rats, decreased statistically in all the test groups after 14 days (Table 4). This decrease is significantly more pronounced with *J. curcas* oilseeds cake than those of *A. floribunda*.

**Table 2:** Nutritional parameters

	**High fat diet**	**Control**	***A. floribunda***	***J. curcas***
Body gain (g)	65 ± 3.08^c^	49.20 ± 3.03^b^	33.49 ± 4.75^a^	29.20 ± 6.36^a^
Food intake (g)	302.60 ± 9.63 ^b^	339.20 ± 8.40^c^	230.80 ± 10.45^a^	238.80 ± 9.40^a^
Water intake (mL)	415.80 ± 13.42 ^d^	555.60 ± 15.22^c^	323 ± 9.42^b^	380.60 ± 16.43^a^
Food efficiency (%)	20.09 ± 0.46 ^b^	11.96 ± 0.64^a^	11.60 ± 0.32^a^	12.22 ± 0.57^a^

Data are presented as mean ± SD (n = 5); mean values within a line with different superscript letters are statistically different (*p* < 0.05).

**Table 3:** Effect of seeds oil-cake on lipid profiles (in brackets represent % of change)

		**High fat diet**	**Control**	***A. floribunda***	***J. curcas***
Plasma	TC (mg /dL)	223.91 ± 20.4^a^		194.65 ± 6.85^b^	182.31 ± 6.59^b^
		(31.71 ± 4.30)	170 ± 5.44^c^	(13.06 ± 0.96)	(18.57 ± 1.3)
	TG (mg/dL)	228.03 ± 16.32^a^		158.42 ± 4.57^c^	197.42 ± 5.48^b^
		(47.17 ± 3.04)	156.22 ± 4.36^c^	(30.52 ± 1.52)	(13.42 ± 2.32)
Liver (post mitochondrial fraction (PMF)	TC (mg/dL)	227.30 ± 15.76^a^		182.9 ± 4.5^c^	199.00 ± 6.1^b^
		(39.02 ± 3.70)	163.5 ± 3.61^d^	(19.53 ± 2.05)	(12.45 ± 1.85)
	TG (mg/dL)	252.91 ± 11.33^a^		361.41 ± 7.76^b^	356.68 ± 8.52^b^
		(41.03 ± 4.21)	177.16 ± 2.93^c^	(42.90 ± 3.60)	(41.03 ± 2.36)

Values in the same row with identical superscripts are not significantly different (*p* < 0.05). % change was calculated using formula % change = [(Tt - Tc)/Tc] × 100, Tt = values of treated group, Tc = values of respective control group; Data are presented as mean ± SD (n = 5); mean values within a line with different superscript letters are statistically different (*p* < 0.05); TC = Total Cholesterol; TG = Triglycerides.

**Table 4:** Effect of seeds oil-cake on the blood glucose level (mg/dL) (in brackets represent % of change)

	**High fat diet**	**Control**	***A. floribunda***	***J. curcas***
Day 0	67.40 ± 12.8^a^	65.80 ± 5.08^a^	64.40 ± 5.20^a^	65.40 ± 9.24^a^
Day 14	96.80 ± 3.82^a^	62.20 ± 3.66^b^	80.5 ± 4.81^d^	67.22 ± 8.13^c^
	(55.62 ± 7.53)		(16.83 ± 1.17)	(30.57 ± 3.21)

Values in the same row with identical superscripts are not significantly different (*p* < 0.05). % change was calculated using formula % change = [(Tt - Tc)/Tc] × 100, Tt = values of treated group, Tc = values of respective control group; Data are presented as mean ± SD (n = 5); Mean values within a line with different superscript letters are statistically different (*p* < 0.05).

## Discussion

The objectives of this research were to study the protein profile and antioxidant properties of oilseed cake generated from *A. floribunda* and *J. curcas,* as well as their effects on weight gain and serum lipid levels in experimental rats placed on a high fat diet. The solubilization of storage proteins in oilseed cake according to Nasri and Triki [15] method and their quantification using the Bradford [16] method show that *A. floribunda* was poorer in proteins and richer in dietary fibre when compared to conventional oilseed cakes which generally contain 35–45% of dried weight [25]. Another particularity was that glutelins were the major protein family in *J. curcas*, while in *A. floribunda* glutelins were at a non-detectable level. This result contrasted with findings by Rabetafika et al. [25] who found that globulins are the major protein family (58–68%) of flax oilseed cake. The level of dietary fibre obtained for *J. curcas* (24.47%) in this work is different from 10.12% reported earlier by Akintayo [26] working on cultivars from Nigeria. The observed differences may be due to various possible factors such as genetic and environmental factors.

From Table 1, it was evident that the different solvents used for the extraction of phenolic compounds from *A. floribunda* and *J. curcas* oilseed cake, had different abilities to extract substances from these oilseed cake. In general, the extraction of phenolic compounds from *A. floribunda* and *J. curcas* oilseed cake with ethanol was found to be the most effective. These findings are not in agreement with those of Matthaüs [27] and Terpinc et al*.*[28] who found that extraction of phenols in various oilseed cakes with water was most effective as compared to alcohol and acid extraction.

Four antioxidant assays, namely Ferric Reducing Antioxidant Potential (FRAP), DPPH · radical scavenging activity, ABTS Free-Radical-Scavenging and ferrous ion-chelating ability were performed in order to explore the antioxidant potential of the oilseed cakes of *A. floribunda* and *J. curcas*. Generally, extracts from *A. floribunda* oilseed cake demonstrated greater reducing power, antiradical power and iron-chelating capacity. It was noticed that the antioxidant potential of *A. floribunda* oilseed cake in DPPH assay was linearly correlated to its total phenolic compounds. These results are in accordance with the report that antioxidant activity increases proportionally with the polyphenol content [29,30]. Phenolic compounds are important phyto constituents and have potential against different diseases because of their antioxidant property [31]. Radical scavenging via hydrogen atom donation by phenols is believed to be the predominant mechanism of antioxidant action [32]. However, no relationship was found between total phenol content and antioxidant efficiencies in *J. curcas* oilseed cake, suggesting that phenolic compounds are not the only contributors to the antioxidant activities of the *J. curcas* defatted cakes [28]. Furthermore, this observation are in line with those of Karadag et al*.*[33] who think that antioxidant and scavenging activities are hydroxyl functions content dependent.

The effect of oilseed cake of *A. floribunda* and *J. curcas* on the lipid profile and blood glucose level of rats placed on high fat diet suggested that they contributed to the regulation of serum TG and TC levels, blood glucose level and decreased dietary intake. These properties could be attributed to the presence of dietary fibres, phenolic compounds and storage proteins on oilseed cake. Generally, dietary fibres have a hypocholesterolaemic effect; modulate blood glucose response and increase satiety [34]. Furthermore, Marambe et al. [35] demonstrated the cholesterol-lowering property of flaxseed proteins by their bile acid binding ability. In the liver, the TC level decreased whereas the TG amount increased significantly. Liver is the main site of lipogenesis. Furthermore, the influence of food fatty acid composition on lipogenesis is well established [36]. The TG level which remained very high could be a consequence of the high fat consumed food.

## Conclusions

The proteins group profile, antioxidant properties and total phenolic content of *A. floribunda* and *J. curcas* oilseed cake differed significantly from each other. This investigation also indicates that oilseed cake of *A. floribunda* and *J. curcas* possess hypolipidaemic effect and regulate blood glucose level. Additional studies are needed to characterize the mechanisms involved and toxicological effect, as it is well known that some *J. curcas* varieties seeds are highly toxic to some animal species due to the presence of toxins and antinutrient components. The present study supports the view that some plants are promising sources of natural antioxidants and are lipid-lowering.

### Ethical considerations

The studies were conducted in accordance with the internationally accepted principles for laboratory animal use and care as found in the United States guidelines (United States National Institutes for Health publication n° 85–23 revised in 1985) and approved by the Cameroon National Ethics Committee.

## Competing interest

The authors declare that they have no competing interests.

## Authors’ contributions

TB conceived the project, participated in protein purification and supervised the work all through, JEKN participated in plant collection, protein purification and biological tests, ALW participated in phenols extraction and biological tests, NYN participated in work design and drafted the manuscript. All the authors proofread and approved the manuscript before submission.

## Pre-publication history

The pre-publication history for this paper can be accessed here:

http://www.biomedcentral.com/1472-6882/13/352/prepub
